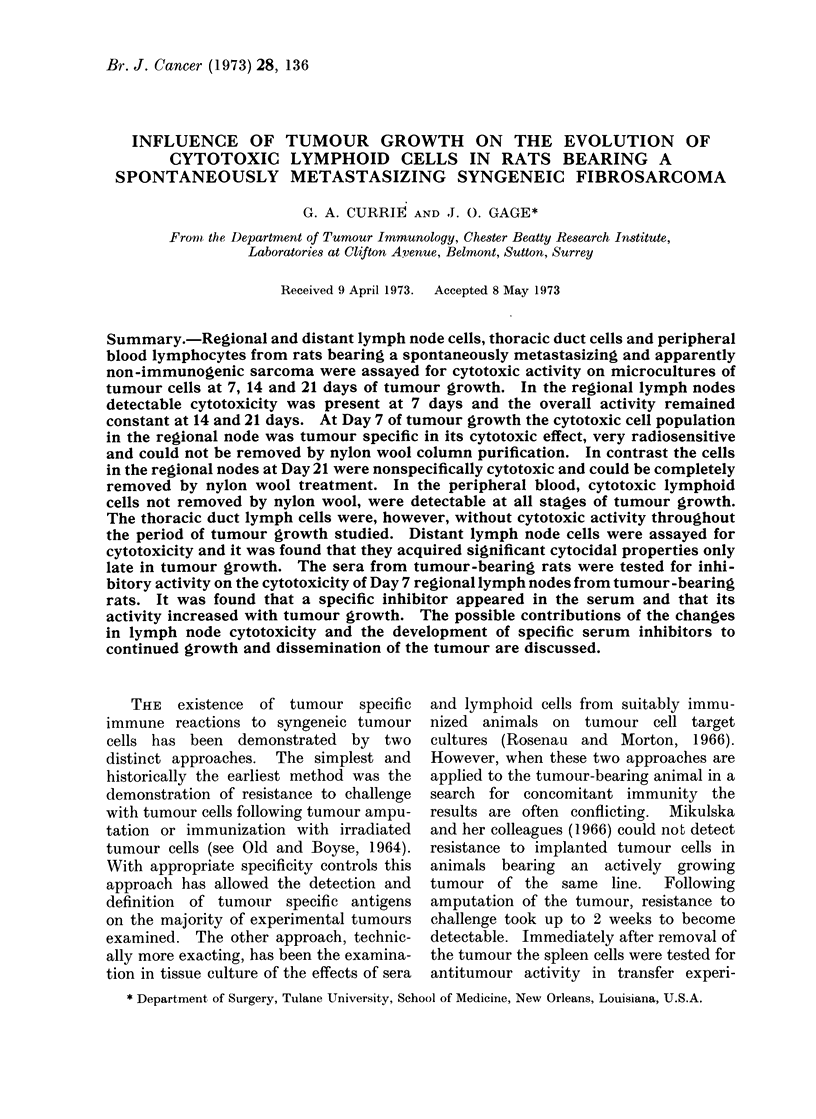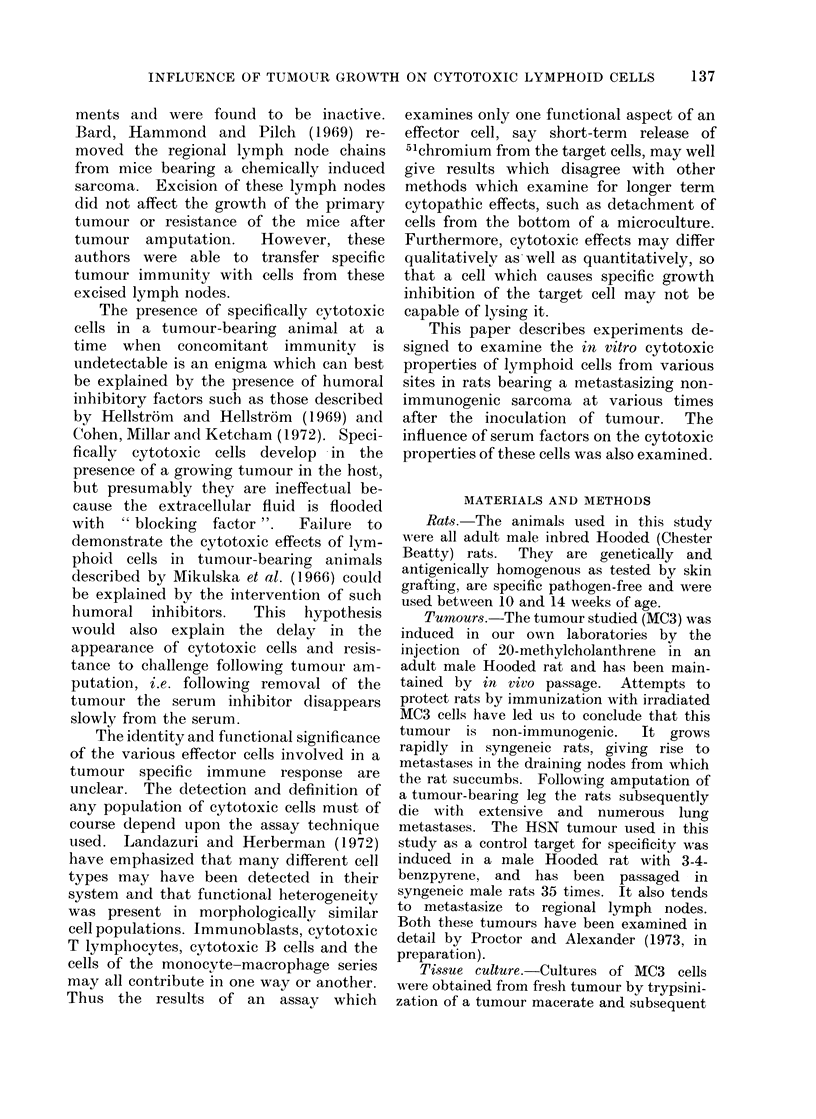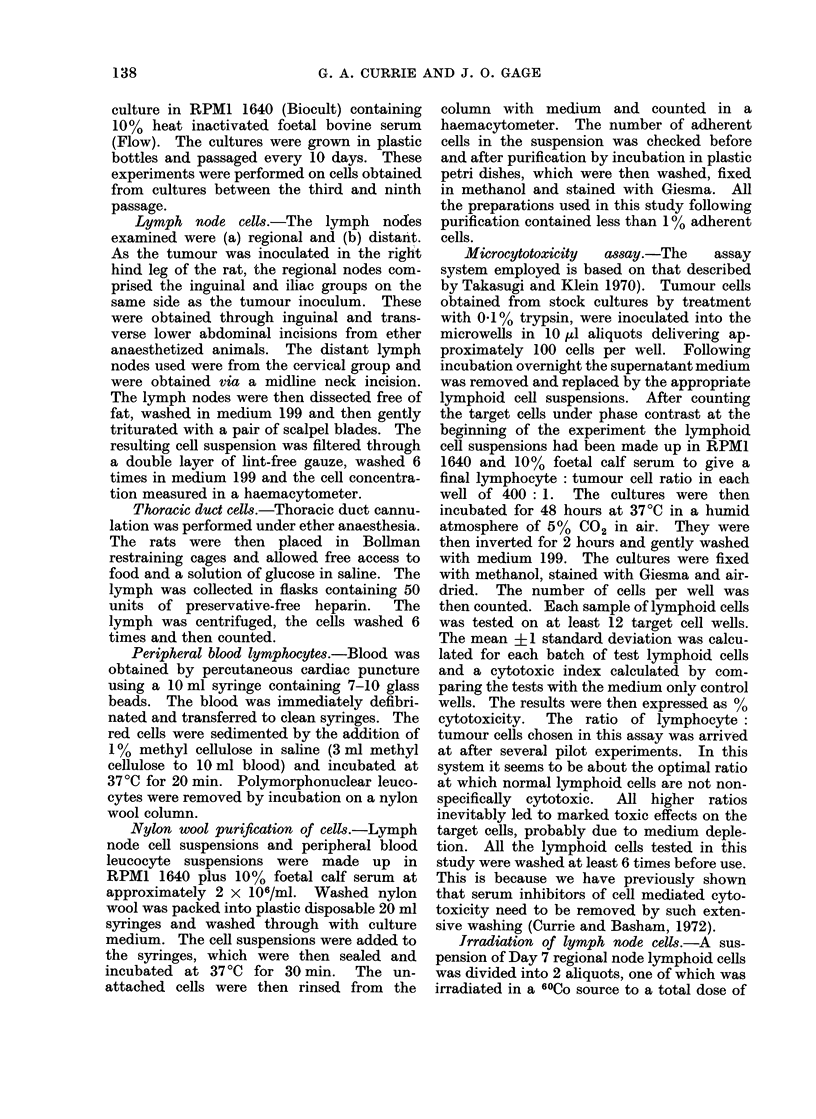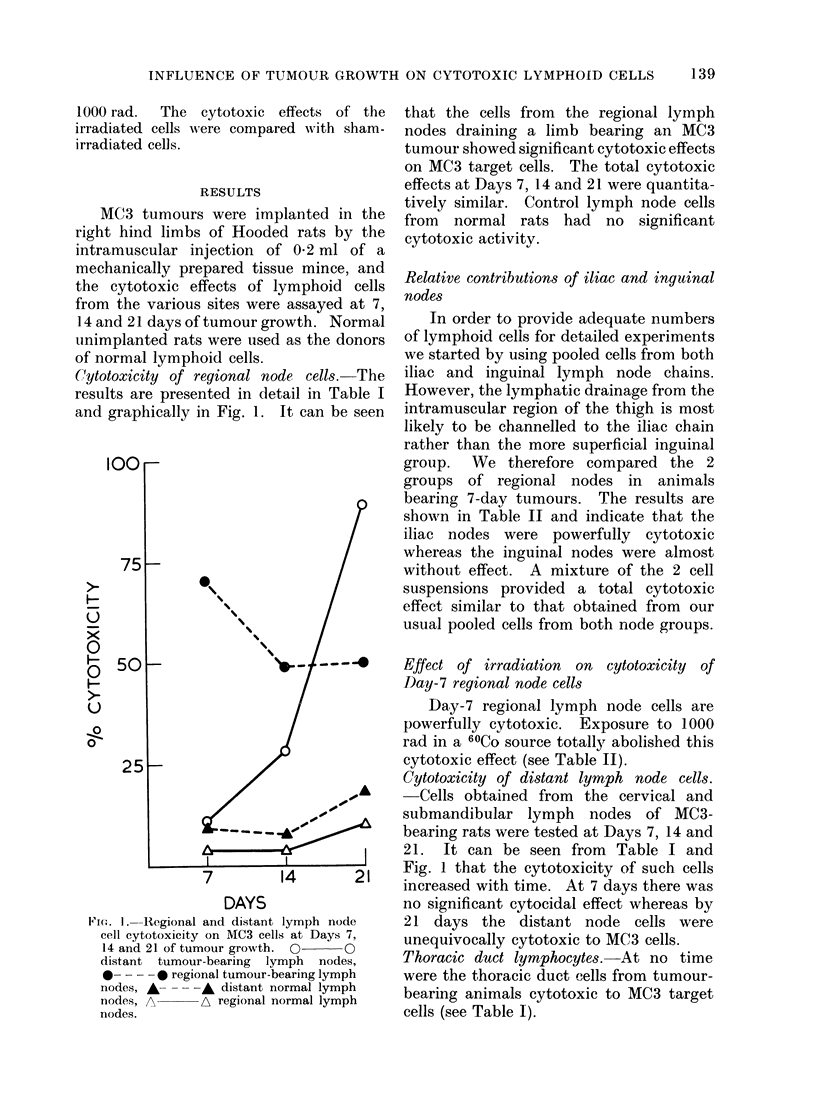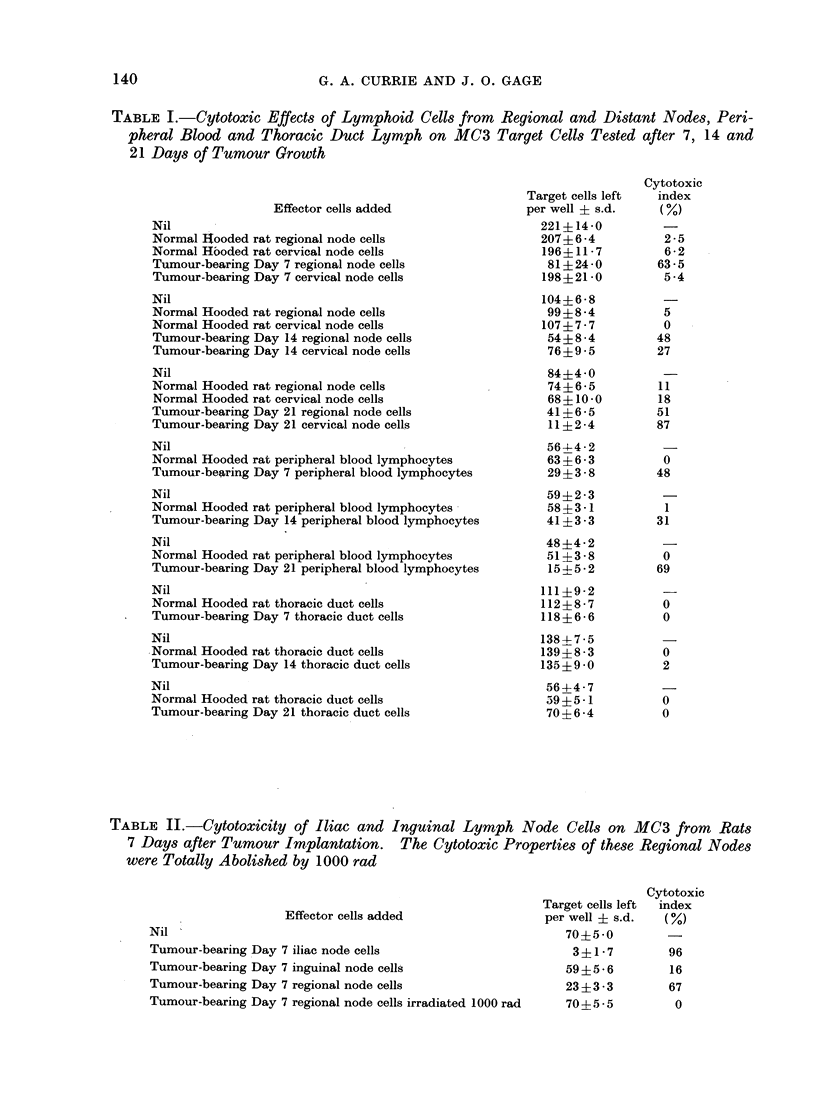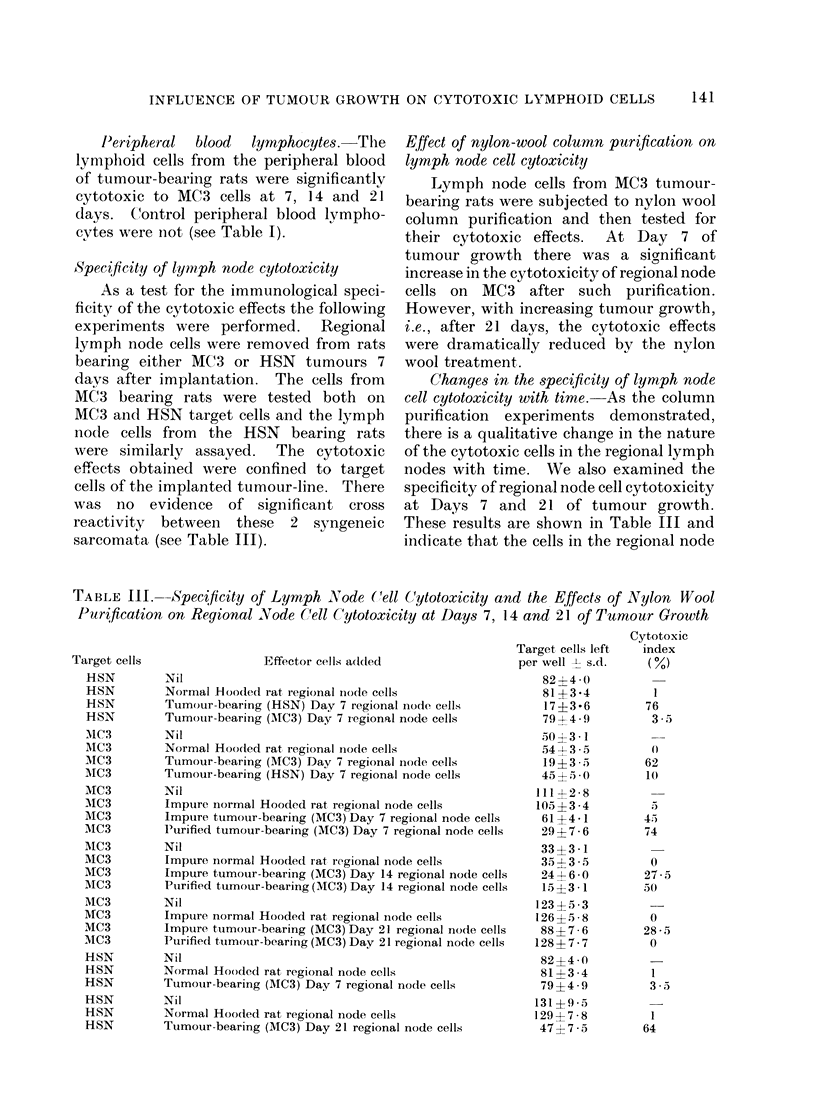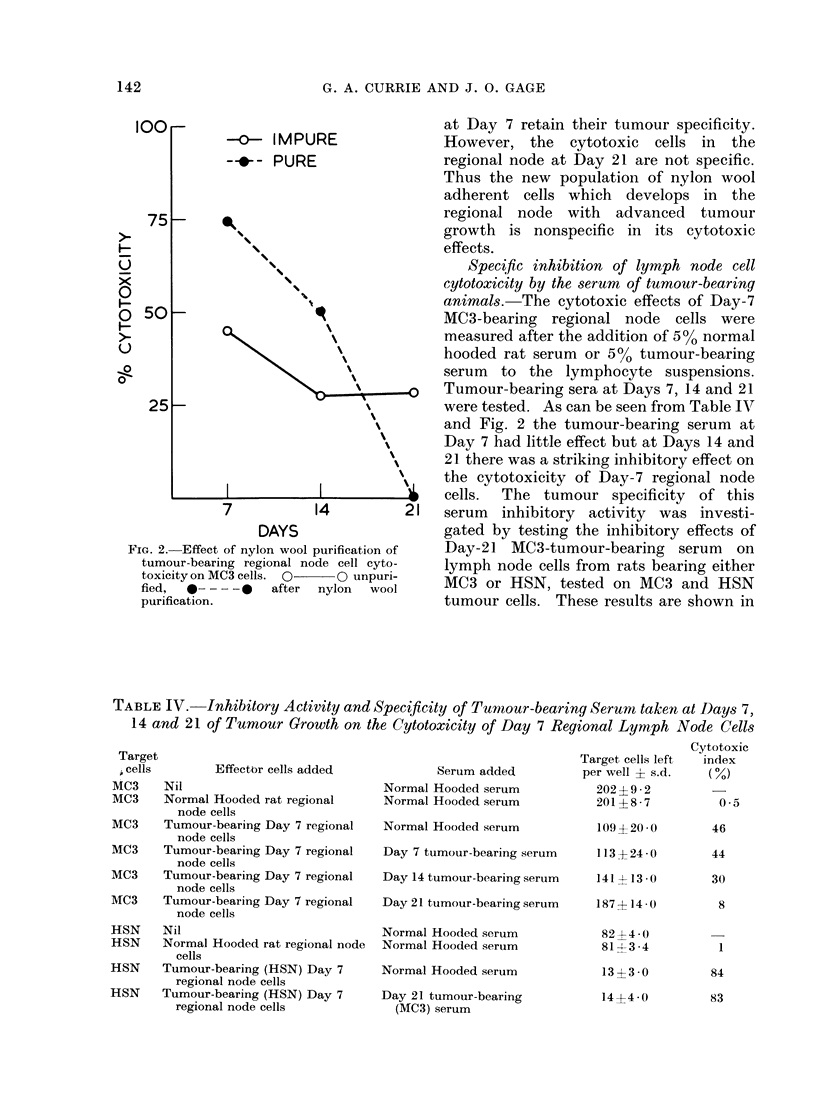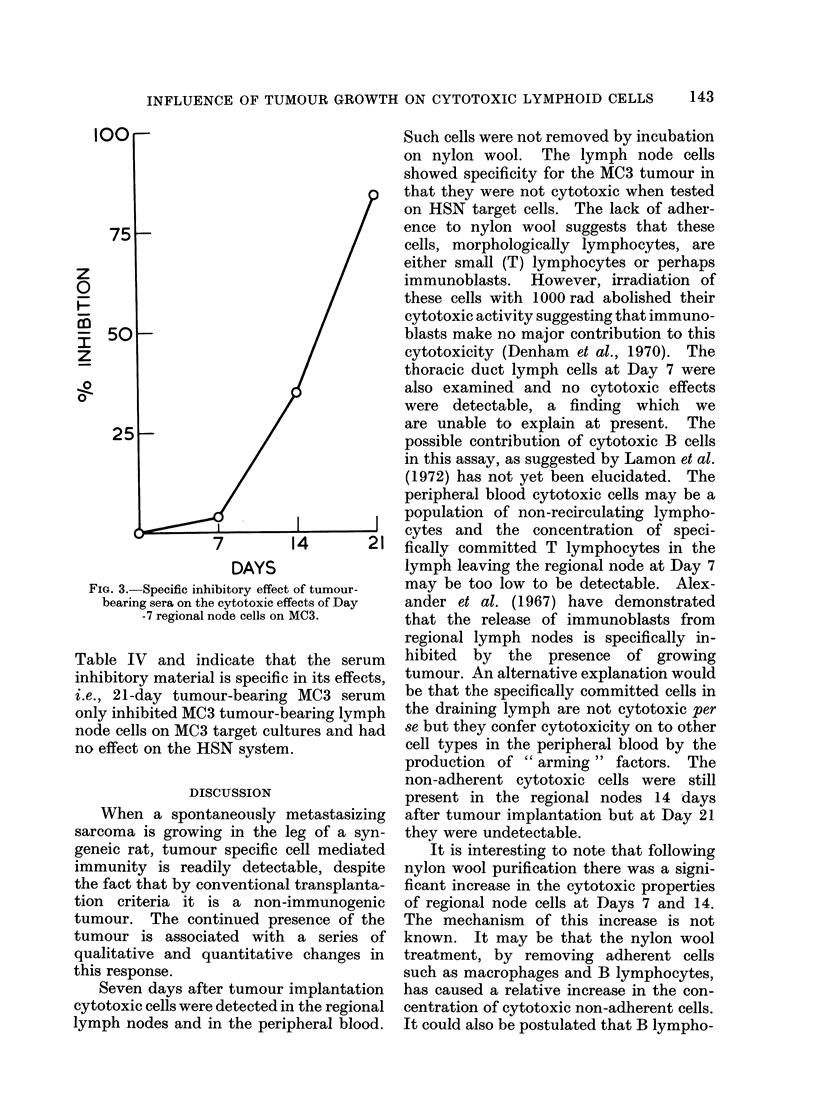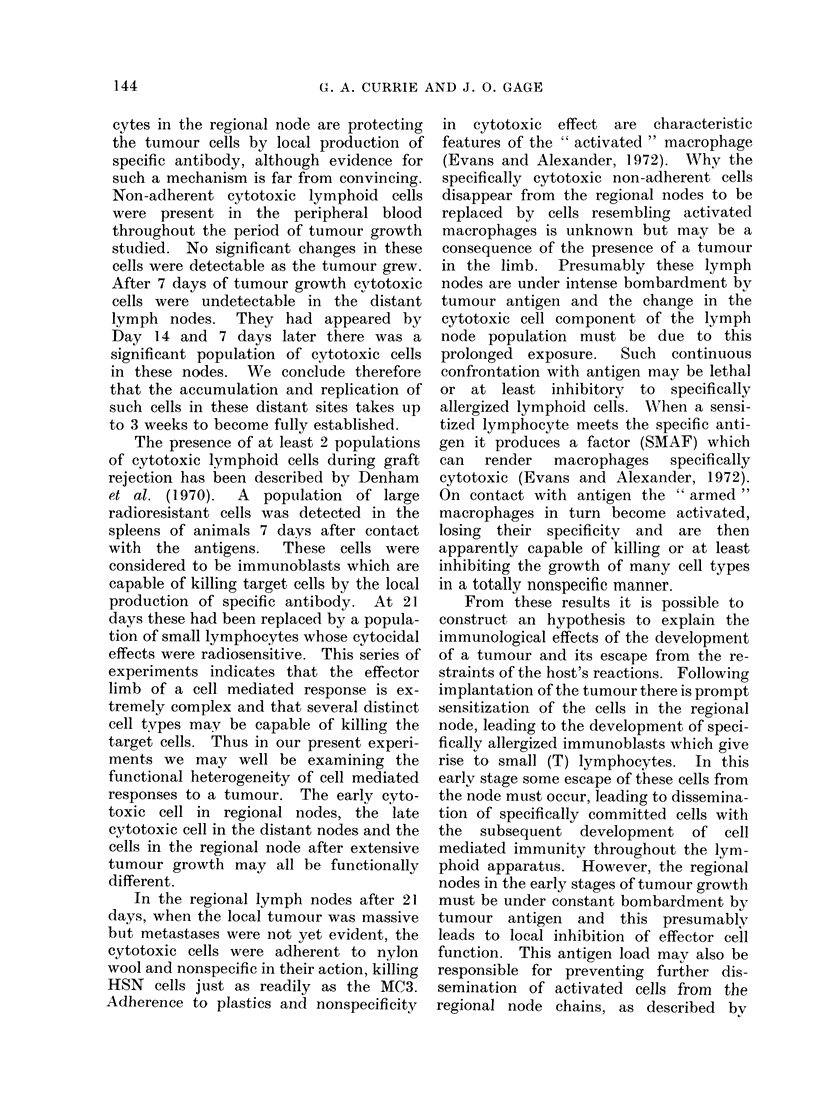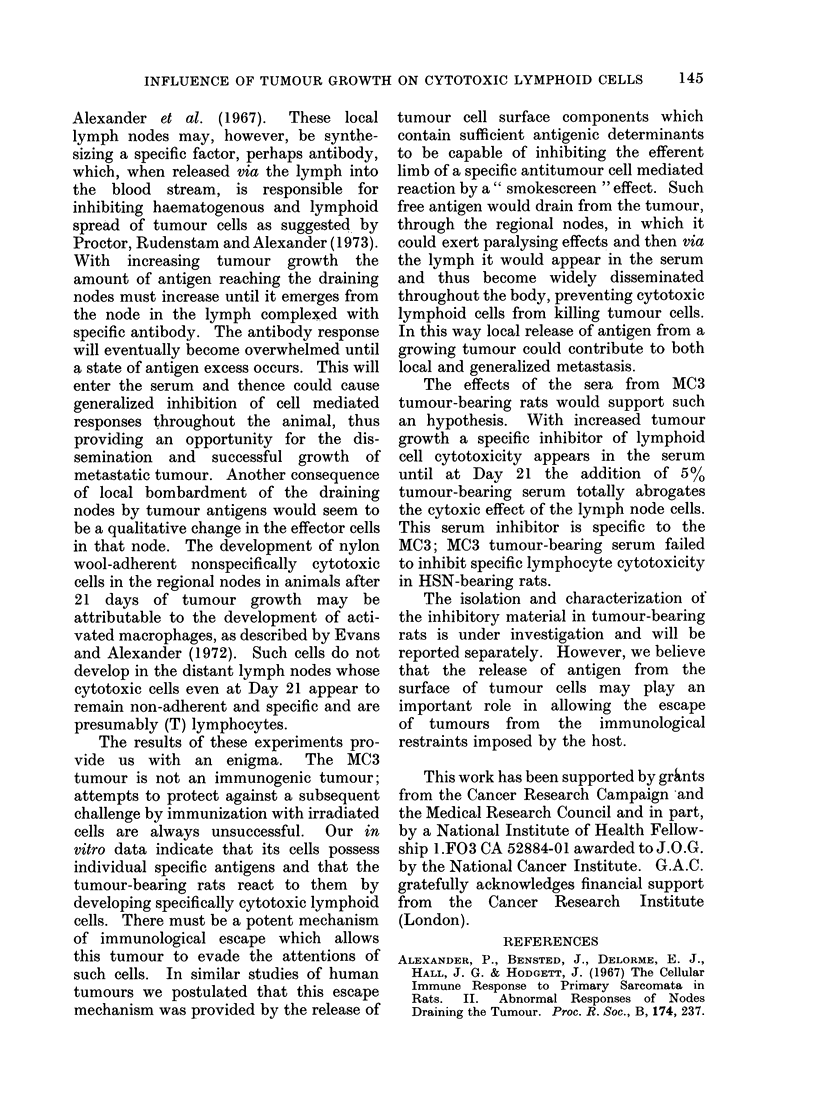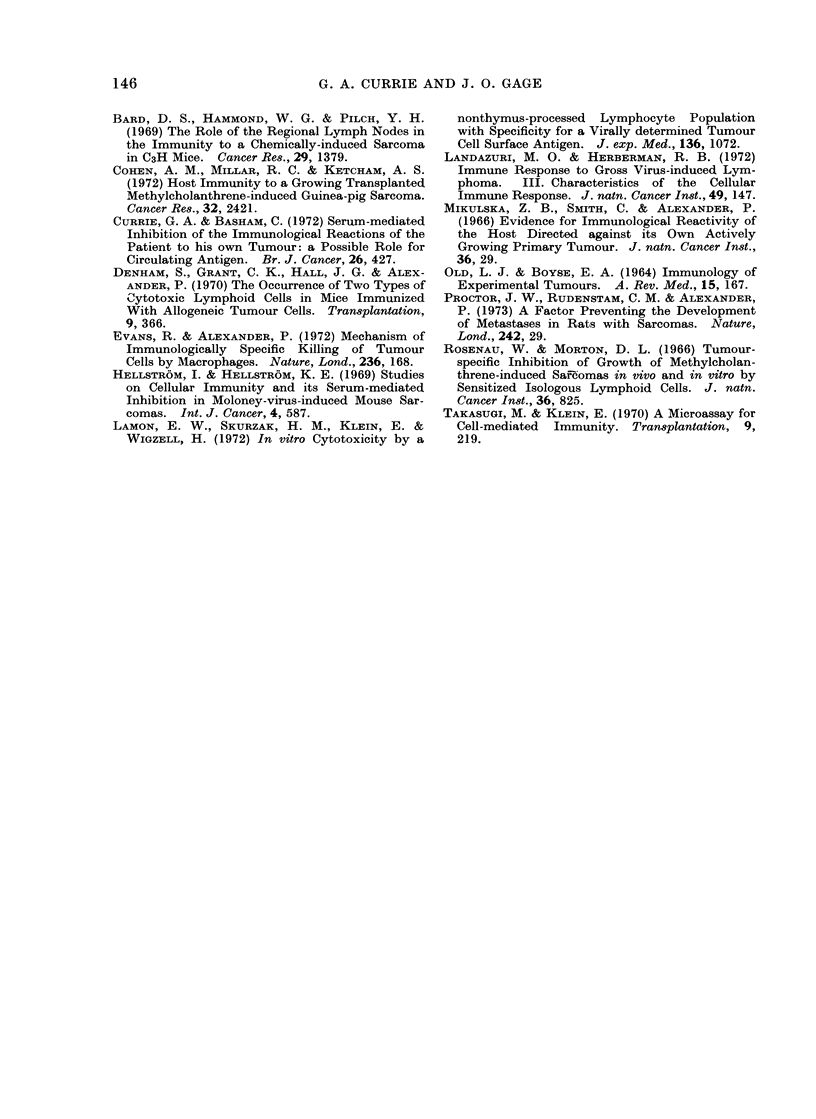# Influence of Tumour Growth on the Evolution of Cytotoxic Lymphoid Cells in Rats Bearing a Spontaneously Metastasizing Syngeneic Fibrosarcoma

**DOI:** 10.1038/bjc.1973.131

**Published:** 1973-08

**Authors:** G. A. Currie, J. O. Gage

## Abstract

Regional and distant lymph node cells, thoracic duct cells and peripheral blood lymphocytes from rats bearing a spontaneously metastasizing and apparently non-immunogenic sarcoma were assayed for cytotoxic activity on microcultures of tumour cells at 7, 14 and 21 days of tumour growth. In the regional lymph nodes detectable cytotoxicity was present at 7 days and the overall activity remained constant at 14 and 21 days. At Day 7 of tumour growth the cytotoxic cell population in the regional node was tumour specific in its cytotoxic effect, very radiosensitive and could not be removed by nylon wool column purification. In contrast the cells in the regional nodes at Day 21 were nonspecifically cytotoxic and could be completely removed by nylon wool treatment. In the peripheral blood, cytotoxic lymphoid cells not removed by nylon wool, were detectable at all stages of tumour growth. The thoracic duct lymph cells were, however, without cytotoxic activity throughout the period of tumour growth studied. Distant lymph node cells were assayed for cytotoxicity and it was found that they acquired significant cytocidal properties only late in tumour growth. The sera from tumour-bearing rats were tested for inhibitory activity on the cytotoxicity of Day 7 regional lymph nodes from tumour-bearing rats. It was found that a specific inhibitor appeared in the serum and that its activity increased with tumour growth. The possible contributions of the changes in lymph node cytotoxicity and the development of specific serum inhibitors to continued growth and dissemination of the tumour are discussed.


*Br. J. Cancer* (1973) **28**, 136

### INFLUENCE OF TUMOUR GROWTH ON THE EVOLUTION OF CYTOTOXIC LYMPHOID CELLS IN RATS BEARING A SPONTANEOUSLY METASTASIZING SYNGENEIC FIBROSARCOMA

G. A. CURRIE AND J. O. GAGE*

*From the Department of Tumour Immunology, Chester Beatty Research Institute, Laboratories at Clifton Avenue, Belmont, Sutton, Surrey*

Received 9 April 1973. Accepted 8 May 1973

**Summary.—Regional and distant lymph node cells, thoracic duct cells and peripheral blood lymphocytes from rats bearing a spontaneously metastasizing and apparently non-immunogenic sarcoma were assayed for cytotoxic activity on microcultures of tumour cells at 7, 14 and 21 days of tumour growth. In the regional lymph nodes detectable cytotoxicity was present at 7 days and the overall activity remained constant at 14 and 21 days. At Day 7 of tumour growth the cytotoxic cell population in the regional node was tumour specific in its cytotoxic effect, very radiosensitive and could not be removed by nylon wool column purification. In contrast the cells in the regional nodes at Day 21 were nonspecifically cytotoxic and could be completely removed by nylon wool treatment. In the peripheral blood, cytotoxic lymphoid cells not removed by nylon wool, were detectable at all stages of tumour growth. The thoracic duct lymph cells were, however, without cytotoxic activity throughout the period of tumour growth studied. Distant lymph node cells were assayed for cytotoxicity and it was found that they acquired significant cytocidal properties only late in tumour growth. The sera from tumour-bearing rats were tested for inhibitory activity on the cytotoxicity of Day 7 regional lymph nodes from tumour-bearing rats. It was found that a specific inhibitor appeared in the serum and that its activity increased with tumour growth. The possible contributions of the changes in lymph node cytotoxicity and the development of specific serum inhibitors to continued growth and dissemination of the tumour are discussed.**

THE existence of tumour specific immune reactions to syngeneic tumour cells has been demonstrated by two distinct approaches. The simplest and historically the earliest method was the demonstration of resistance to challenge with tumour cells following tumour amputation or immunization with irradiated tumour cells (see Old and Boyse, 1964). With appropriate specificity controls this approach has allowed the detection and definition of tumour specific antigens on the majority of experimental tumours examined. The other approach, technically more exacting, has been the examination in tissue culture of the effects of sera and lymphoid cells from suitably immunized animals on tumour cell target cultures (Rosenau and Morton, 1966). However, when these two approaches are applied to the tumour-bearing animal in a search for concomitant immunity the results are often conflicting. Mikulska and her colleagues (1966) could not detect resistance to implanted tumour cells in animals bearing an actively growing tumour of the same line. Following amputation of the tumour, resistance to challenge took up to 2 weeks to become detectable. Immediately after removal of the tumour the spleen cells were tested for antitumour activity in transfer experi-

* Department of Surgery, Tulane University, School of Medicine, New Orleans, Louisiana, U.S.A.

ments and were found to be inactive. Bard, Hammond and Pilch (1969) removed the regional lymph node chains from mice bearing a chemically induced sarcoma. Excision of these lymph nodes did not affect the growth of the primary tumour or resistance of the mice after tumour amputation. However, these authors were able to transfer specific tumour immunity with cells from these excised lymph nodes.

The presence of specifically cytotoxic cells in a tumour-bearing animal at a time when concomitant immunity is undetectable is an enigma which can best be explained by the presence of humoral inhibitory factors such as those described by Hellström and Hellström (1969) and Cohen, Millar and Ketcham (1972). Specifically cytotoxic cells develop in the presence of a growing tumour in the host, but presumably they are ineffectual because the extracellular fluid is flooded with " blocking factor ". Failure to demonstrate the cytotoxic effects of lymphoid cells in tumour-bearing animals described by Mikulska *et al.* (1966) could be explained by the intervention of such humoral inhibitors. This hypothesis would also explain the delay in the appearance of cytotoxic cells and resistance to challenge following tumour amputation, *i.e.* following removal of the tumour the serum inhibitor disappears slowly from the serum.

The identity and functional significance of the various effector cells involved in a tumour specific immune response are unclear. The detection and definition of any population of cytotoxic cells must of course depend upon the assay technique used. Landazuri and Herberman (1972) have emphasized that many different cell types may have been detected in their system and that functional heterogeneity was present in morphologically similar cell populations. Immunoblasts, cytotoxic T lymphocytes, cytotoxic B cells and the cells of the monocyte–macrophage series may all contribute in one way or another. Thus the results of an assay which examines only one functional aspect of an effector cell, say short-term release of $^{51}$chromium from the target cells, may well give results which disagree with other methods which examine for longer term cytopathic effects, such as detachment of cells from the bottom of a microculture. Furthermore, cytotoxic effects may differ qualitatively as well as quantitatively, so that a cell which causes specific growth inhibition of the target cell may not be capable of lysing it.

This paper describes experiments designed to examine the *in vitro* cytotoxic properties of lymphoid cells from various sites in rats bearing a metastasizing non-immunogenic sarcoma at various times after the inoculation of tumour. The influence of serum factors on the cytotoxic properties of these cells was also examined.

### MATERIALS AND METHODS

*Rats.*—The animals used in this study were all adult male inbred Hooded (Chester Beatty) rats. They are genetically and antigenically homogenous as tested by skin grafting, are specific pathogen-free and were used between 10 and 14 weeks of age.

*Tumours.*—The tumour studied (MC3) was induced in our own laboratories by the injection of 20-methylcholanthrene in an adult male Hooded rat and has been maintained by *in vivo* passage. Attempts to protect rats by immunization with irradiated MC3 cells have led us to conclude that this tumour is non-immunogenic. It grows rapidly in syngeneic rats, giving rise to metastases in the draining nodes from which the rat succumbs. Following amputation of a tumour-bearing leg the rats subsequently die with extensive and numerous lung metastases. The HSN tumour used in this study as a control target for specificity was induced in a male Hooded rat with 3-4-benzpyrene, and has been passaged in syngeneic male rats 35 times. It also tends to metastasize to regional lymph nodes. Both these tumours have been examined in detail by Proctor and Alexander (1973, in preparation).

*Tissue culture.*—Cultures of MC3 cells were obtained from fresh tumour by trypsinization of a tumour macerate and subsequent culture in RPM1 1640 (Biocult) containing 10% heat inactivated foetal bovine serum (Flow). The cultures were grown in plastic bottles and passaged every 10 days. These experiments were performed on cells obtained from cultures between the third and ninth passage.

*Lymph node cells.*—The lymph nodes examined were (a) regional and (b) distant. As the tumour was inoculated in the right hind leg of the rat, the regional nodes comprised the inguinal and iliac groups on the same side as the tumour inoculum. These were obtained through inguinal and transverse lower abdominal incisions from ether anaesthetized animals. The distant lymph nodes used were from the cervical group and were obtained *via* a midline neck incision. The lymph nodes were then dissected free of fat, washed in medium 199 and then gently triturated with a pair of scalpel blades. The resulting cell suspension was filtered through a double layer of lint-free gauze, washed 6 times in medium 199 and the cell concentration measured in a haemacytometer.

*Thoracic duct cells.*—Thoracic duct cannulation was performed under ether anaesthesia. The rats were then placed in Bollman restraining cages and allowed free access to food and a solution of glucose in saline. The lymph was collected in flasks containing 50 units of preservative-free heparin. The lymph was centrifuged, the cells washed 6 times and then counted.

*Peripheral blood lymphocytes.*—Blood was obtained by percutaneous cardiac puncture using a 10 ml syringe containing 7–10 glass beads. The blood was immediately defibrinated and transferred to clean syringes. The red cells were sedimented by the addition of 1% methyl cellulose in saline (3 ml methyl cellulose to 10 ml blood) and incubated at 37°C for 20 min. Polymorphonuclear leucocytes were removed by incubation on a nylon wool column.

*Nylon wool purification of cells.*—Lymph node cell suspensions and peripheral blood leucocyte suspensions were made up in RPM1 1640 plus 10% foetal calf serum at approximately $2 \times 10^6$/ml. Washed nylon wool was packed into plastic disposable 20 ml syringes and washed through with culture medium. The cell suspensions were added to the syringes, which were then sealed and incubated at 37°C for 30 min. The unattached cells were then rinsed from the column with medium and counted in a haemacytometer. The number of adherent cells in the suspension was checked before and after purification by incubation in plastic petri dishes, which were then washed, fixed in methanol and stained with Giesma. All the preparations used in this study following purification contained less than 1% adherent cells.

*Microcytotoxicity assay.*—The assay system employed is based on that described by Takasugi and Klein 1970). Tumour cells obtained from stock cultures by treatment with 0·1% trypsin, were inoculated into the microwells in 10 $\mu$l aliquots delivering approximately 100 cells per well. Following incubation overnight the supernatant medium was removed and replaced by the appropriate lymphoid cell suspensions. After counting the target cells under phase contrast at the beginning of the experiment the lymphoid cell suspensions had been made up in RPM1 1640 and 10% foetal calf serum to give a final lymphocyte : tumour cell ratio in each well of 400 : 1. The cultures were then incubated for 48 hours at 37°C in a humid atmosphere of 5% $CO_2$ in air. They were then inverted for 2 hours and gently washed with medium 199. The cultures were fixed with methanol, stained with Giesma and air-dried. The number of cells per well was then counted. Each sample of lymphoid cells was tested on at least 12 target cell wells. The mean $\pm 1$ standard deviation was calculated for each batch of test lymphoid cells and a cytotoxic index calculated by comparing the tests with the medium only control wells. The results were then expressed as % cytotoxicity. The ratio of lymphocyte : tumour cells chosen in this assay was arrived at after several pilot experiments. In this system it seems to be about the optimal ratio at which normal lymphoid cells are not non-specifically cytotoxic. All higher ratios inevitably led to marked toxic effects on the target cells, probably due to medium depletion. All the lymphoid cells tested in this study were washed at least 6 times before use. This is because we have previously shown that serum inhibitors of cell mediated cytotoxicity need to be removed by such extensive washing (Currie and Basham, 1972).

*Irradiation of lymph node cells.*—A suspension of Day 7 regional node lymphoid cells was divided into 2 aliquots, one of which was irradiated in a $^{60}$Co source to a total dose of

1000 rad. The cytotoxic effects of the irradiated cells were compared with sham-irradiated cells.

RESULTS

MC3 tumours were implanted in the right hind limbs of Hooded rats by the intramuscular injection of 0·2 ml of a mechanically prepared tissue mince, and the cytotoxic effects of lymphoid cells from the various sites were assayed at 7, 14 and 21 days of tumour growth. Normal unimplanted rats were used as the donors of normal lymphoid cells.

*Cytotoxicity of regional node cells.*—The results are presented in detail in Table I and graphically in Fig. 1. It can be seen

FIG. 1.—Regional and distant lymph node cell cytotoxicity on MC3 cells at Days 7, 14 and 21 of tumour growth. ○———○ distant tumour-bearing lymph nodes, ●- - - -● regional tumour-bearing lymph nodes, ▲- - - -▲ distant normal lymph nodes, △———△ regional normal lymph nodes.

that the cells from the regional lymph nodes draining a limb bearing an MC3 tumour showed significant cytotoxic effects on MC3 target cells. The total cytotoxic effects at Days 7, 14 and 21 were quantitatively similar. Control lymph node cells from normal rats had no significant cytotoxic activity.

*Relative contributions of iliac and inguinal nodes*

In order to provide adequate numbers of lymphoid cells for detailed experiments we started by using pooled cells from both iliac and inguinal lymph node chains. However, the lymphatic drainage from the intramuscular region of the thigh is most likely to be channelled to the iliac chain rather than the more superficial inguinal group. We therefore compared the 2 groups of regional nodes in animals bearing 7-day tumours. The results are shown in Table II and indicate that the iliac nodes were powerfully cytotoxic whereas the inguinal nodes were almost without effect. A mixture of the 2 cell suspensions provided a total cytotoxic effect similar to that obtained from our usual pooled cells from both node groups.

*Effect of irradiation on cytotoxicity of Day-7 regional node cells*

Day-7 regional lymph node cells are powerfully cytotoxic. Exposure to 1000 rad in a $^{60}$Co source totally abolished this cytotoxic effect (see Table II).

*Cytotoxicity of distant lymph node cells.*—Cells obtained from the cervical and submandibular lymph nodes of MC3-bearing rats were tested at Days 7, 14 and 21. It can be seen from Table I and Fig. 1 that the cytotoxicity of such cells increased with time. At 7 days there was no significant cytocidal effect whereas by 21 days the distant node cells were unequivocally cytotoxic to MC3 cells.

*Thoracic duct lymphocytes.*—At no time were the thoracic duct cells from tumour-bearing animals cytotoxic to MC3 target cells (see Table I).

TABLE I.—*Cytotoxic Effects of Lymphoid Cells from Regional and Distant Nodes, Peripheral Blood and Thoracic Duct Lymph on MC3 Target Cells Tested after 7, 14 and 21 Days of Tumour Growth*

| Effector cells added | Target cells left per well ± s.d. | Cytotoxic index (%) |
|---|---|---|
| Nil | 221±14·0 | — |
| Normal Hooded rat regional node cells | 207±6·4 | 2·5 |
| Normal Hooded rat cervical node cells | 196±11·7 | 6·2 |
| Tumour-bearing Day 7 regional node cells | 81±24·0 | 63·5 |
| Tumour-bearing Day 7 cervical node cells | 198±21·0 | 5·4 |
| Nil | 104±6·8 | — |
| Normal Hooded rat regional node cells | 99±8·4 | 5 |
| Normal Hooded rat cervical node cells | 107±7·7 | 0 |
| Tumour-bearing Day 14 regional node cells | 54±8·4 | 48 |
| Tumour-bearing Day 14 cervical node cells | 76±9·5 | 27 |
| Nil | 84±4·0 | — |
| Normal Hooded rat regional node cells | 74±6·5 | 11 |
| Normal Hooded rat cervical node cells | 68±10·0 | 18 |
| Tumour-bearing Day 21 regional node cells | 41±6·5 | 51 |
| Tumour-bearing Day 21 cervical node cells | 11±2·4 | 87 |
| Nil | 56±4·2 | — |
| Normal Hooded rat peripheral blood lymphocytes | 63±6·3 | 0 |
| Tumour-bearing Day 7 peripheral blood lymphocytes | 29±3·8 | 48 |
| Nil | 59±2·3 | — |
| Normal Hooded rat peripheral blood lymphocytes | 58±3·1 | 1 |
| Tumour-bearing Day 14 peripheral blood lymphocytes | 41±3·3 | 31 |
| Nil | 48±4·2 | — |
| Normal Hooded rat peripheral blood lymphocytes | 51±3·8 | 0 |
| Tumour-bearing Day 21 peripheral blood lymphocytes | 15±5·2 | 69 |
| Nil | 111±9·2 | — |
| Normal Hooded rat thoracic duct cells | 112±8·7 | 0 |
| Tumour-bearing Day 7 thoracic duct cells | 118±6·6 | 0 |
| Nil | 138±7·5 | — |
| Normal Hooded rat thoracic duct cells | 139±8·3 | 0 |
| Tumour-bearing Day 14 thoracic duct cells | 135±9·0 | 2 |
| Nil | 56±4·7 | — |
| Normal Hooded rat thoracic duct cells | 59±5·1 | 0 |
| Tumour-bearing Day 21 thoracic duct cells | 70±6·4 | 0 |

TABLE II.—*Cytotoxicity of Iliac and Inguinal Lymph Node Cells on MC3 from Rats 7 Days after Tumour Implantation. The Cytotoxic Properties of these Regional Nodes were Totally Abolished by 1000 rad*

| Effector cells added | Target cells left per well ± s.d. | Cytotoxic index (%) |
|---|---|---|
| Nil | 70±5·0 | — |
| Tumour-bearing Day 7 iliac node cells | 3±1·7 | 96 |
| Tumour-bearing Day 7 inguinal node cells | 59±5·6 | 16 |
| Tumour-bearing Day 7 regional node cells | 23±3·3 | 67 |
| Tumour-bearing Day 7 regional node cells irradiated 1000 rad | 70±5·5 | 0 |

*Peripheral blood lymphocytes.*—The lymphoid cells from the peripheral blood of tumour-bearing rats were significantly cytotoxic to MC3 cells at 7, 14 and 21 days. Control peripheral blood lymphocytes were not (see Table I).

*Specificity of lymph node cytotoxicity*

As a test for the immunological specificity of the cytotoxic effects the following experiments were performed. Regional lymph node cells were removed from rats bearing either MC3 or HSN tumours 7 days after implantation. The cells from MC3 bearing rats were tested both on MC3 and HSN target cells and the lymph node cells from the HSN bearing rats were similarly assayed. The cytotoxic effects obtained were confined to target cells of the implanted tumour-line. There was no evidence of significant cross reactivity between these 2 syngeneic sarcomata (see Table III).

*Effect of nylon-wool column purification on lymph node cell cytoxicity*

Lymph node cells from MC3 tumour-bearing rats were subjected to nylon wool column purification and then tested for their cytotoxic effects. At Day 7 of tumour growth there was a significant increase in the cytotoxicity of regional node cells on MC3 after such purification. However, with increasing tumour growth, *i.e.*, after 21 days, the cytotoxic effects were dramatically reduced by the nylon wool treatment.

*Changes in the specificity of lymph node cell cytotoxicity with time.*—As the column purification experiments demonstrated, there is a qualitative change in the nature of the cytotoxic cells in the regional lymph nodes with time. We also examined the specificity of regional node cell cytotoxicity at Days 7 and 21 of tumour growth. These results are shown in Table III and indicate that the cells in the regional node

TABLE III.—*Specificity of Lymph Node Cell Cytotoxicity and the Effects of Nylon Wool Purification on Regional Node Cell Cytotoxicity at Days* 7, 14 *and* 21 *of Tumour Growth*

| Target cells | Effector cells added | Target cells left per well ± s.d. | Cytotoxic index (%) |
|---|---|---|---|
| HSN | Nil | 82±4·0 | — |
| HSN | Normal Hooded rat regional node cells | 81±3·4 | 1 |
| HSN | Tumour-bearing (HSN) Day 7 regional node cells | 17±3·6 | 76 |
| HSN | Tumour-bearing (MC3) Day 7 regional node cells | 79±4·9 | 3·5 |
| MC3 | Nil | 50±3·1 | — |
| MC3 | Normal Hooded rat regional node cells | 54±3·5 | 0 |
| MC3 | Tumour-bearing (MC3) Day 7 regional node cells | 19±3·5 | 62 |
| MC3 | Tumour-bearing (HSN) Day 7 regional node cells | 45±5·0 | 10 |
| MC3 | Nil | 111±2·8 | — |
| MC3 | Impure normal Hooded rat regional node cells | 105±3·4 | 5 |
| MC3 | Impure tumour-bearing (MC3) Day 7 regional node cells | 61±4·1 | 45 |
| MC3 | Purified tumour-bearing (MC3) Day 7 regional node cells | 29±7·6 | 74 |
| MC3 | Nil | 33±3·1 | — |
| MC3 | Impure normal Hooded rat regional node cells | 35±3·5 | 0 |
| MC3 | Impure tumour-bearing (MC3) Day 14 regional node cells | 24±6·0 | 27·5 |
| MC3 | Purified tumour-bearing (MC3) Day 14 regional node cells | 15±3·1 | 50 |
| MC3 | Nil | 123±5·3 | — |
| MC3 | Impure normal Hooded rat regional node cells | 126±5·8 | 0 |
| MC3 | Impure tumour-bearing (MC3) Day 21 regional node cells | 88±7·6 | 28·5 |
| MC3 | Purified tumour-bearing (MC3) Day 21 regional node cells | 128±7·7 | 0 |
| HSN | Nil | 82±4·0 | — |
| HSN | Normal Hooded rat regional node cells | 81±3·4 | 1 |
| HSN | Tumour-bearing (MC3) Day 7 regional node cells | 79±4·9 | 3·5 |
| HSN | Nil | 131±9·5 | — |
| HSN | Normal Hooded rat regional node cells | 129±7·8 | 1 |
| HSN | Tumour-bearing (MC3) Day 21 regional node cells | 47±7·5 | 64 |

FIG. 2.—Effect of nylon wool purification of tumour-bearing regional node cell cytotoxicity on MC3 cells. ○———○ unpurified, ●– – – –● after nylon wool purification.

at Day 7 retain their tumour specificity. However, the cytotoxic cells in the regional node at Day 21 are not specific. Thus the new population of nylon wool adherent cells which develops in the regional node with advanced tumour growth is nonspecific in its cytotoxic effects.

*Specific inhibition of lymph node cell cytotoxicity by the serum of tumour-bearing animals.*—The cytotoxic effects of Day-7 MC3-bearing regional node cells were measured after the addition of 5% normal hooded rat serum or 5% tumour-bearing serum to the lymphocyte suspensions. Tumour-bearing sera at Days 7, 14 and 21 were tested. As can be seen from Table IV and Fig. 2 the tumour-bearing serum at Day 7 had little effect but at Days 14 and 21 there was a striking inhibitory effect on the cytotoxicity of Day-7 regional node cells. The tumour specificity of this serum inhibitory activity was investigated by testing the inhibitory effects of Day-21 MC3-tumour-bearing serum on lymph node cells from rats bearing either MC3 or HSN, tested on MC3 and HSN tumour cells. These results are shown in

TABLE IV.—*Inhibitory Activity and Specificity of Tumour-bearing Serum taken at Days* 7, 14 *and* 21 *of Tumour Growth on the Cytotoxicity of Day* 7 *Regional Lymph Node Cells*

| Target cells | Effector cells added | Serum added | Target cells left per well ± s.d. | Cytotoxic index (%) |
|---|---|---|---|---|
| MC3 | Nil | Normal Hooded serum | 202±9·2 | — |
| MC3 | Normal Hooded rat regional node cells | Normal Hooded serum | 201±8·7 | 0·5 |
| MC3 | Tumour-bearing Day 7 regional node cells | Normal Hooded serum | 109±20·0 | 46 |
| MC3 | Tumour-bearing Day 7 regional node cells | Day 7 tumour-bearing serum | 113±24·0 | 44 |
| MC3 | Tumour-bearing Day 7 regional node cells | Day 14 tumour-bearing serum | 141±13·0 | 30 |
| MC3 | Tumour-bearing Day 7 regional node cells | Day 21 tumour-bearing serum | 187±14·0 | 8 |
| HSN | Nil | Normal Hooded serum | 82±4·0 | — |
| HSN | Normal Hooded rat regional node cells | Normal Hooded serum | 81±3·4 | 1 |
| HSN | Tumour-bearing (HSN) Day 7 regional node cells | Normal Hooded serum | 13±3·0 | 84 |
| HSN | Tumour-bearing (HSN) Day 7 regional node cells | Day 21 tumour-bearing (MC3) serum | 14±4·0 | 83 |

FIG. 3.—Specific inhibitory effect of tumour-bearing sera on the cytotoxic effects of Day -7 regional node cells on MC3.

Table IV and indicate that the serum inhibitory material is specific in its effects, *i.e.*, 21-day tumour-bearing MC3 serum only inhibited MC3 tumour-bearing lymph node cells on MC3 target cultures and had no effect on the HSN system.

### DISCUSSION

When a spontaneously metastasizing sarcoma is growing in the leg of a syngeneic rat, tumour specific cell mediated immunity is readily detectable, despite the fact that by conventional transplantation criteria it is a non-immunogenic tumour. The continued presence of the tumour is associated with a series of qualitative and quantitative changes in this response.

Seven days after tumour implantation cytotoxic cells were detected in the regional lymph nodes and in the peripheral blood. Such cells were not removed by incubation on nylon wool. The lymph node cells showed specificity for the MC3 tumour in that they were not cytotoxic when tested on HSN target cells. The lack of adherence to nylon wool suggests that these cells, morphologically lymphocytes, are either small (T) lymphocytes or perhaps immunoblasts. However, irradiation of these cells with 1000 rad abolished their cytotoxic activity suggesting that immunoblasts make no major contribution to this cytotoxicity (Denham *et al.*, 1970). The thoracic duct lymph cells at Day 7 were also examined and no cytotoxic effects were detectable, a finding which we are unable to explain at present. The possible contribution of cytotoxic B cells in this assay, as suggested by Lamon *et al.* (1972) has not yet been elucidated. The peripheral blood cytotoxic cells may be a population of non-recirculating lymphocytes and the concentration of specifically committed T lymphocytes in the lymph leaving the regional node at Day 7 may be too low to be detectable. Alexander *et al.* (1967) have demonstrated that the release of immunoblasts from regional lymph nodes is specifically inhibited by the presence of growing tumour. An alternative explanation would be that the specifically committed cells in the draining lymph are not cytotoxic *per se* but they confer cytotoxicity on to other cell types in the peripheral blood by the production of "arming" factors. The non-adherent cytotoxic cells were still present in the regional nodes 14 days after tumour implantation but at Day 21 they were undetectable.

It is interesting to note that following nylon wool purification there was a significant increase in the cytotoxic properties of regional node cells at Days 7 and 14. The mechanism of this increase is not known. It may be that the nylon wool treatment, by removing adherent cells such as macrophages and B lymphocytes, has caused a relative increase in the concentration of cytotoxic non-adherent cells. It could also be postulated that B lymphocytes in the regional node are protecting the tumour cells by local production of specific antibody, although evidence for such a mechanism is far from convincing. Non-adherent cytotoxic lymphoid cells were present in the peripheral blood throughout the period of tumour growth studied. No significant changes in these cells were detectable as the tumour grew. After 7 days of tumour growth cytotoxic cells were undetectable in the distant lymph nodes. They had appeared by Day 14 and 7 days later there was a significant population of cytotoxic cells in these nodes. We conclude therefore that the accumulation and replication of such cells in these distant sites takes up to 3 weeks to become fully established.

The presence of at least 2 populations of cytotoxic lymphoid cells during graft rejection has been described by Denham *et al.* (1970). A population of large radioresistant cells was detected in the spleens of animals 7 days after contact with the antigens. These cells were considered to be immunoblasts which are capable of killing target cells by the local production of specific antibody. At 21 days these had been replaced by a population of small lymphocytes whose cytocidal effects were radiosensitive. This series of experiments indicates that the effector limb of a cell mediated response is extremely complex and that several distinct cell types may be capable of killing the target cells. Thus in our present experiments we may well be examining the functional heterogeneity of cell mediated responses to a tumour. The early cytotoxic cell in regional nodes, the late cytotoxic cell in the distant nodes and the cells in the regional node after extensive tumour growth may all be functionally different.

In the regional lymph nodes after 21 days, when the local tumour was massive but metastases were not yet evident, the cytotoxic cells were adherent to nylon wool and nonspecific in their action, killing HSN cells just as readily as the MC3. Adherence to plastics and nonspecificity in cytotoxic effect are characteristic features of the " activated " macrophage (Evans and Alexander, 1972). Why the specifically cytotoxic non-adherent cells disappear from the regional nodes to be replaced by cells resembling activated macrophages is unknown but may be a consequence of the presence of a tumour in the limb. Presumably these lymph nodes are under intense bombardment by tumour antigen and the change in the cytotoxic cell component of the lymph node population must be due to this prolonged exposure. Such continuous confrontation with antigen may be lethal or at least inhibitory to specifically allergized lymphoid cells. When a sensitized lymphocyte meets the specific antigen it produces a factor (SMAF) which can render macrophages specifically cytotoxic (Evans and Alexander, 1972). On contact with antigen the " armed " macrophages in turn become activated, losing their specificity and are then apparently capable of killing or at least inhibiting the growth of many cell types in a totally nonspecific manner.

From these results it is possible to construct an hypothesis to explain the immunological effects of the development of a tumour and its escape from the restraints of the host's reactions. Following implantation of the tumour there is prompt sensitization of the cells in the regional node, leading to the development of specifically allergized immunoblasts which give rise to small (T) lymphocytes. In this early stage some escape of these cells from the node must occur, leading to dissemination of specifically committed cells with the subsequent development of cell mediated immunity throughout the lymphoid apparatus. However, the regional nodes in the early stages of tumour growth must be under constant bombardment by tumour antigen and this presumably leads to local inhibition of effector cell function. This antigen load may also be responsible for preventing further dissemination of activated cells from the regional node chains, as described by

Alexander *et al.* (1967). These local lymph nodes may, however, be synthesizing a specific factor, perhaps antibody, which, when released *via* the lymph into the blood stream, is responsible for inhibiting haematogenous and lymphoid spread of tumour cells as suggested by Proctor, Rudenstam and Alexander (1973). With increasing tumour growth the amount of antigen reaching the draining nodes must increase until it emerges from the node in the lymph complexed with specific antibody. The antibody response will eventually become overwhelmed until a state of antigen excess occurs. This will enter the serum and thence could cause generalized inhibition of cell mediated responses throughout the animal, thus providing an opportunity for the dissemination and successful growth of metastatic tumour. Another consequence of local bombardment of the draining nodes by tumour antigens would seem to be a qualitative change in the effector cells in that node. The development of nylon wool-adherent nonspecifically cytotoxic cells in the regional nodes in animals after 21 days of tumour growth may be attributable to the development of activated macrophages, as described by Evans and Alexander (1972). Such cells do not develop in the distant lymph nodes whose cytotoxic cells even at Day 21 appear to remain non-adherent and specific and are presumably (T) lymphocytes.

The results of these experiments provide us with an enigma. The MC3 tumour is not an immunogenic tumour; attempts to protect against a subsequent challenge by immunization with irradiated cells are always unsuccessful. Our *in vitro* data indicate that its cells possess individual specific antigens and that the tumour-bearing rats react to them by developing specifically cytotoxic lymphoid cells. There must be a potent mechanism of immunological escape which allows this tumour to evade the attentions of such cells. In similar studies of human tumours we postulated that this escape mechanism was provided by the release of tumour cell surface components which contain sufficient antigenic determinants to be capable of inhibiting the efferent limb of a specific antitumour cell mediated reaction by a " smokescreen " effect. Such free antigen would drain from the tumour, through the regional nodes, in which it could exert paralysing effects and then *via* the lymph it would appear in the serum and thus become widely disseminated throughout the body, preventing cytotoxic lymphoid cells from killing tumour cells. In this way local release of antigen from a growing tumour could contribute to both local and generalized metastasis.

The effects of the sera from MC3 tumour-bearing rats would support such an hypothesis. With increased tumour growth a specific inhibitor of lymphoid cell cytotoxicity appears in the serum until at Day 21 the addition of 5% tumour-bearing serum totally abrogates the cytoxic effect of the lymph node cells. This serum inhibitor is specific to the MC3; MC3 tumour-bearing serum failed to inhibit specific lymphocyte cytotoxicity in HSN-bearing rats.

The isolation and characterization of the inhibitory material in tumour-bearing rats is under investigation and will be reported separately. However, we believe that the release of antigen from the surface of tumour cells may play an important role in allowing the escape of tumours from the immunological restraints imposed by the host.

This work has been supported by grants from the Cancer Research Campaign and the Medical Research Council and in part, by a National Institute of Health Fellowship 1.FO3 CA 52884-01 awarded to J.O.G. by the National Cancer Institute. G.A.C. gratefully acknowledges financial support from the Cancer Research Institute (London).

### REFERENCES

Alexander, P., Bensted, J., Delorme, E. J., Hall, J. G. & Hodgett, J. (1967) The Cellular Immune Response to Primary Sarcomata in Rats. II. Abnormal Responses of Nodes Draining the Tumour. *Proc. R. Soc.*, B, **174**, 237.

BARD, D. S., HAMMOND, W. G. & PILCH, Y. H. (1969) The Role of the Regional Lymph Nodes in the Immunity to a Chemically-induced Sarcoma in C3H Mice. *Cancer Res.*, **29**, 1379.

COHEN, A. M., MILLAR, R. C. & KETCHAM, A. S. (1972) Host Immunity to a Growing Transplanted Methylcholanthrene-induced Guinea-pig Sarcoma. *Cancer Res.*, **32**, 2421.

CURRIE, G. A. & BASHAM, C. (1972) Serum-mediated Inhibition of the Immunological Reactions of the Patient to his own Tumour: a Possible Role for Circulating Antigen. *Br. J. Cancer*, **26**, 427.

DENHAM, S., GRANT, C. K., HALL, J. G. & ALEXANDER, P. (1970) The Occurrence of Two Types of Cytotoxic Lymphoid Cells in Mice Immunized With Allogeneic Tumour Cells. *Transplantation*, **9**, 366.

EVANS, R. & ALEXANDER, P. (1972) Mechanism of Immunologically Specific Killing of Tumour Cells by Macrophages. *Nature, Lond.*, **236**, 168.

HELLSTRÖM, I. & HELLSTRÖM, K. E. (1969) Studies on Cellular Immunity and its Serum-mediated Inhibition in Moloney-virus-induced Mouse Sarcomas. *Int. J. Cancer*, **4**, 587.

LAMON, E. W., SKURZAK, H. M., KLEIN, E. & WIGZELL, H. (1972) *In vitro* Cytotoxicity by a nonthymus-processed Lymphocyte Population with Specificity for a Virally determined Tumour Cell Surface Antigen. *J. exp. Med.*, **136**, 1072.

LANDAZURI, M. O. & HERBERMAN, R. B. (1972) Immune Response to Gross Virus-induced Lymphoma. III. Characteristics of the Cellular Immune Response. *J. natn. Cancer Inst.*, **49**, 147.

MIKULSKA, Z. B., SMITH, C. & ALEXANDER, P. (1966) Evidence for Immunological Reactivity of the Host Directed against its Own Actively Growing Primary Tumour. *J. natn. Cancer Inst.*, **36**, 29.

OLD, L. J. & BOYSE, E. A. (1964) Immunology of Experimental Tumours. *A. Rev. Med.*, **15**, 167.

PROCTOR, J. W., RUDENSTAM, C. M. & ALEXANDER, P. (1973) A Factor Preventing the Development of Metastases in Rats with Sarcomas. *Nature, Lond.*, **242**, 29.

ROSENAU, W. & MORTON, D. L. (1966) Tumour-specific Inhibition of Growth of Methylcholanthrene-induced Sarcomas *in vivo* and *in vitro* by Sensitized Isologous Lymphoid Cells. *J. natn. Cancer Inst.*, **36**, 825.

TAKASUGI, M. & KLEIN, E. (1970) A Microassay for Cell-mediated Immunity. *Transplantation*, **9**, 219.